# Intima-media thickness and arterial function in obese and non-obese children

**DOI:** 10.1186/s40608-016-0081-9

**Published:** 2016-01-09

**Authors:** Heidi Weberruß, Raphael Pirzer, Birgit Böhm, Robert Dalla Pozza, Heinrich Netz, Renate Oberhoffer

**Affiliations:** 1 Institute of Preventive Pediatrics, Technische Universität München, Georg-Brauchle-Ring 60/62, Campus D, 80992 Munich, Germany; 2 Department of Pediatric Cardiology, Ludwig-Maximilians-University, Marchioninistraße 15, 81377 Munich, Germany

**Keywords:** Arterial stiffness, Cardiovascular disease, Pediatrics, Prevention, Ultrasound

## Abstract

**Background:**

Obesity is an independent cardiovascular risk factor that contributes to the development of atherosclerosis. Subclinical forms of the disease can be assessed via sonographic measurement of carotid intima-media thickness (cIMT) and distensibility – both may already be altered in childhood. As childhood obesity increases to an alarming extent, this study compares vascular data of obese with normal weight boys and girls to investigate the influence of obesity on cIMT and distensibility of the carotid arteries.

**Methods:**

cIMT and distensibility of 46 obese children (27 girls) aged 7–17 years were compared with measures of 46 sex- and age-matched normal weight controls. cIMT and distensibility were measured by B- and M-mode ultrasound and expressed as standard deviation scores (SDS). Arterial distensibility was defined by arterial compliance (AC), elastic modulus (Ep), stiffness index β (β), and local pulse wave velocity β (PWV β).

**Results:**

Obese girls had significantly stiffer arteries compared with normal weight girls (Ep SDS 0.64 ± 1.24 vs. 0 ± 1.06, β SDS 0.6 ± 1.17 vs. -0.01 ± 1.06 *p* < .01, PWV β 0.54 ± 1.2 vs. -0.12 ± 1.05 *p* < .05). No significant differences were observed for boys. In multiregression analysis, BMI significantly influenced Ep, β and PWV β but not cIMT and AC.

**Conclusions:**

Obese girls seemed to be at higher cardiovascular risk than boys, expressed by stiffer arteries in obese girls compared with normal weight girls. Overall, BMI negatively influenced parameters of arterial stiffness (Ep, β and PWV β) but not compliance or cIMT.

## Background

Childhood obesity increases rapidly in modern developed countries, which leads to adverse effects on children’s health [1]. Regarding the cardiovascular system, childhood obesity is claimed as metabolic fundament for atherosclerosis in adulthood [2, 3]. Subclinical indicators of atherosclerosis, like an increased carotid intima-media thickness (cIMT) and impaired arterial distensibility, can already be detected in childhood via ultrasound. Most studies report a significantly increased cIMT in obese children and adolescents compared with normal weight controls [4]. For arterial distensibility, findings are contradictory. Several studies report impaired parameters, hence increased arterial stiffness and reduced compliance [5–7], while others observed lower stiffness and higher compliance in obese children compared with normal weight controls [8–11].

So far, none of these studies addressed sex differences of cIMT and arterial distensibility in obese children. In the normal weight population, girls are reported to have higher arterial stiffness parameters than boys during pre-puberty, as well as women after menopause compared with men [12–14]. According to these findings, this study’s purpose was to investigate the influence of obesity on cIMT and distensibility in boys and girls.

## Methods

Data collection was part of the prevention project “Sternstunden der Gesundheit”, a prospective cross-sectional study conducted from October 2012 to July 2013 in the area of Berchtesgadener Land, Germany. 1017 healthy children (483 boys/534 girls) aged 7–18 were examined to establish reference values for cIMT and parameters of arterial distensibility [15]. Children had no history of chronic disease or signs of acute infection. The study was approved by the ethics committee of the Technische Universität München (5490/12), written informed consent was obtained from parents and from parents and children ≥ 14 years.

Out of the total study population, *n* = 46 children (27 girls) were obese, according to German reference values which define obesity as BMI SDS above the 97^th^ percentile [16]. Obese children were compared with *n* = 46 sex- and age-matched normal weight controls.

Anthropometric measurements were performed by trained staff according to standardized guidelines [17]. Body weight was measured without shoes, wearing light clothes to the nearest 0.1 kg, body height was measured with a stadiometer (seca 799, seca, Hamburg, Germany), standing upright without shoes to the nearest 0.1 cm. Body-Mass-Index (BMI) was calculated from the ratio of mass (kg) to height^2^ (m^2^).

cIMT and distensibility were assessed using semi-automated B- and M-Mode ultrasound, (ProSound Alpha 6, Aloka/Hitachi Medical Systems GmbH, Wiesbaden, Germany) with a high frequency linear array probe (5–13 MHz). After 15 min rest patients were examined in supine position, the neck slightly extended and their head turned 45° opposite the site being scanned. cIMT was measured in B-Mode according to the Mannheim consensus [18] on the common carotid artery (CCA) far wall, 1 cm proximal to the bulb at end-diastolic moment. The cardiac cycle was simultaneously controlled with a 3 lead ECG. Of each subject, four measurements were performed, two on the left and two on the right CCA and calculated as average mean value of four measurements.

Distensibility was assessed in real time M-Mode with high precision vascular echo tracking at the same location than cIMT. Two tracking gates were placed on the CCA near and far wall which followed vessel wall motion, thereby calculating diameter change during heart cycles. Four video loops of at least five heart cycles were stored, two for the left and two for the right CCA. Distensibility parameters were calculated as average mean value of four measurements. As distensibility is pressure dependent [19], blood pressure (BP) was measured oscillometrically on the left arm (Mobil-O-Graph®, I.E.M., Stolberg, Germany) after 10 min rest, and applied in the calculation. Hypertension was defined as BP SDS above the 95^th^ percentile according to German reference values [20].

CCA distensibility was defined by arterial compliance (AC), elastic modulus (Ep), stiffness index β (β) and local pulse wave velocity (PWV β) according to following formulae [21]:

$$ AC = \pi \left({D}_{ma{x}^2}-{D}_{mi{n}^2}\right)/\left[4\left(B{P}_{max}-B{P}_{min}\right)\right] $$

AC defines the ability of an artery to increase its volume in response to a given increase in blood pressure and is the inverse of arterial stiffness. It is calculated from changes in blood vessel cross-sectional area (D) and BP.

$$ Ep = \left(B{P}_{max}-B{P}_{min}\right)/\left[\left({D}_{max}-{D}_{min}\right)/{D}_{min}\right] $$

Ep increases with increasing vessel stiffness. The parameter is affected by BP.

$$ \beta = ln\left(B{P}_{max}\ /\ B{P}_{min}\right)/\left[\left({D}_{max}-{D}_{min}\right)/{D}_{min}\right] $$

Like Ep, β increases with increasing vessel stiffness, but is low in BP dependency.

$$ PWV\beta = \sqrt{\left(\left(\beta *B{P}_{min}\right)/\left(2\rho \right)\right)} $$

In general, PWV is the speed at which the forward pressure wave is transmitted from the aorta through the vascular tree. In this study, PWV β is assessed as CCA local pulse wave velocity, calculated from β.

All measurements were performed by two experienced examiners. The coefficient of variation (CV) between both examiners, assessed in 27 subjects, was 4.79 for cIMT, and 3.54 % for distensibility, calculated as average CV of AC (4.47 %), Ep (3.42 %), β (4.92 %) and PWV β (1.37 %).

BMI, BP, cIMT and distensibility measures were expressed as sex- and age-dependent standard deviation scores (SDS), calculated as follows:

$$ SDS = \frac{{\left(\frac{x}{M}\right)}^{L-1}}{L\ x\ S}\;\mathrm{f}\mathrm{o}\mathrm{r}\;\mathrm{L}\ne 0\kern0.24em \mathrm{o}\mathrm{r}\kern0.24em SDS = \frac{ \ln \left(\frac{x}{M}\right)}{S}\;\mathrm{f}\mathrm{o}\mathrm{r}\;\mathrm{L}=0 $$

Data was analyzed using IBM SPSS statistics for Windows, version 21.0 (SPSS, Inc., Chicago, IL, USA). After testing for normal distribution, differences between obese and normal weight children were analyzed by independent samples *t*-test or Mann–Whitney *U*-test, for boys and girls separately. The independent influence of BMI and BP on vascular data was analyzed by multivariate stepwise linear regression, and the association with BMI by bivariate correlation, controlled for sex, age, and BP. A P-value of <0.05 was considered to be statistically significant.

## Results and discussion

This subsample of 46 obese children and 46 sex- and age-matched controls was part of the prevention project “Sternstunden der Gesundheit”, a cross-sectional prospective study to establish reference values for cIMT and distensibility measures [15]. Out of the total study population (*n* = 1017 children), measurements of intima-media thickness and arterial distensibility were performed in 656 children (353 girls) of which 46 children (7 %) were obese (27 girls). Anthropometric and vascular data for normal weight and obese boys and girls are displayed in Table 1. Weight, BMI, and BMI SDS differed significantly between normal weight and obese boys and girls (p < .01), there were no significant differences in age, height and BP. Elevated systolic BP levels were present in 24 children (14 girls), elevated diastolic BP levels in 5 (1 girl) and both, elevated systolic and diastolic levles in 2 children (1 girl). 14 children with elevated systolic BP were obese (8 girls) and 10 were normal weight (6 girls). 4 children with elevated diastolic BP were obese (1 girl), and both subjects with elevated systolic and diastolic BP levels were obese.

**Table 1:** Anthropometric and arterial status of normal weight and obese boys and girls

	Male (19/19)						Female (27/27)					
	Normal weight			Obese			Normal weight			Obese		
AGE [yrs]	11.82	±	2.51	11.81	±	2.51	12.68	±	2.84	12.68	±	2.84
HEIGHT [cm]	149.83	±	14.38	157.73	±	16.34	153.71	±	14.62	155.45	±	10.12
WEIGHT [kg]	40.14	±	13.35**	69.91	±	21.99**	42.34	±	12.19**	70.17	±	17.83**
BMI SDS	−0.37	±	0.69**	2.22	±	0.25**	−0.56	±	0.52**	2.35	±	0.5**
BP systolic SDS	0.68	±	1.01	1.23	±	1.05	1.14	±	1.33	1.07	±	1.38
BP diastolic SDS	−0.14	±	1.32	0.55	±	1.02	0.08	±	1.19	0.15	±	1.26
IMT SDS	−0.05	±	0.94	0.03	±	1.39	0.07	±	1.09	0.23	±	0.83
AC SDS	−0.35	±	1.19	0.25	±	0.77	−0.15	±	0.92	0.04	±	1.47
Ep SDS	0.08	±	1.31	0.36	±	1.10	0.00	±	1.06*	0.64	±	1.24*
β SDS	0.23	±	1.28	0.23	±	1.00	−0.01	±	1.06**	0.60	±	1.17**
PWV β SDS	−0.04	±	1.19	0.34	±	1.00	−0.12	±	1.05*	0.54	±	1.2*

*p < .05, **p < .01

cIMT SDS did not differ significantly between normal weight and obese boys and girls. Mean cIMT SDS in normal weight boys were −0.05 ± 0.94 compared with 0.03 ± 1.39 in obese boys and 0.07 ± 1.09 in normal weight girls compared with 0.23 ± 0.83 in obese girls. In boys, distensibility parameters did not differ significantly between normal weight and obese participants. Obese girls, on the contrary had significantly increased stiffness parameters compared with normal weight controls (Table 1).

Multivariate stepwise linear regression revealed no significant independent influence of BMI and BP on cIMT. AC was affected by BP but not BMI. Measures of arterial stiffness (Ep, β and PWV β) were influenced by body dimensions, indicating stiffer arteries at a higher BMI (Table 2). In bivariate correlation, parameters of arterial stiffness were also significantly correlated to BMI after controlling for sex, age, and BP (Ep SDS: *r* = 0.26 β SDS: *r* = 0.25 and PWV β SDS: *r* = 0.25, *p* < .05).

**Table 2:** Stepwise multiregression analysis for measures of arterial distensibility with BMI and BP SDS

	AC SDS R^2^ = 0.43			Ep SDS R^2^ = 0.3			β SDS R^2^ = 0.35			PWV β SDS R^2^ = 0.18		
	Beta ± SE	*β*	*P*	Beta ± SE	*β*	*P*	Beta ± SE	*β*	*P*	Beta ± SE	*β*	*P*
Constant	0.54 ± 0.12		.005	−0.37 ± 0.16		.02	0.19 ± 0.14		.2	−0.3 ± 0.16		.05
BMI SDS				0.18 ± 0.07	0.23	.013	0.16 ± 0.07	0.21	.019	0.17 ± 0.07	0.22	.025
Systolic BP SDS	−0.64 ± 0.08	−0.68	<.001	0.52 ± 0.1	0.54	<.001	0.38 ± 0.09	0.41	<.001	0.32 ± 0.09	0.35	<.001
Diastolic BP SDS	0.48 ± 0.08	0.5	<.001	−0.37 ± 0.1	−0.38	.001	−0.58 ± 0.9	−0.61	<.001			

*BMI* indicates Body Mass Index, *BP* Blood Pressure. All measures are expressed as Standard Deviation Scores

This study compared the vascular status of 46 obese children to 46 age- and sex-matched normal weight controls, separately for boys and girls. Contrary to other studies [4], we did not observe significant higher cIMT values in obese boys and girls. In accordance to findings of Tounian et al. [5], Aggoun et al. [22], and Di Salvo et al. [23] our sample consisted of healthy children with no history of chronic disease. BP levels did not differ significantly between obese and normal weight children, none of the children was diagnosed with manifest hypertension.

The extent of obesity within the sample was 2.22 SDS in boys and 2.35 SDS in girls. Dangardt et al. [8] included obese children with BMI measures > 3 SDS, a significantly higher systolic and significantly lower diastolic BP, and found higher radial artery intimal thickening in obese participants. Giannini et al. [3] included participants with a BMI > 2 SDS and significantly higher systolic and diastolic BP than in lean controls. The coexistence of two risk factors, like hypertension and obesity [24] may lead to a higher increase in cIMT compared to healthy normal weight controls, which can explain non-significant differences in our study with obesity as only risk factor.

Significant differences in parameters of arterial stiffness in contrast to findings on cIMT may be explained with an earlier alteration of an artery’s function compared to its structural status [25]. Ep, β and PWV β were independently affected by BMI to a similar extent (Table 2), indicating stiffer arteries with increasing BMI values. AC in our sample, was higher in obese boys and girls compared to controls but did not reach statistical significance. These results are in accordance with Chalmers et al. [9], Tryggestad et al. [11], and Lurbe et al. [10] who also observed increased compliance in obese children. Dangardt et al. [8] explain this higher compliance as chronic vasodilation caused by initial adaptation of the arterial system to a larger blood volume, generated by increased body fat mass. Tryggestad et al. [11] add that childhood obesity may cause an earlier peak in vascular compliance by obesity related acceleration of pubertal development. Chalmers et al. [9] claim the significantly higher pubertal status in their obese participants to be responsible for higher circulating insulin, leading to chronic vasodilation. According to the authors, this status remains until other risk factors contribute to the development of atherosclerosis – at this point the vessel wall surpasses a point of natural adaptation that results in decreased compliance and increased stiffness.

In this study, stiffness parameters in obese girls differed significantly from those in normal weight sex- and age-matched controls which is not the case in male participants. Marlatt et al. [26] did not find significantly sex differences for arterial stiffness parameters in normal weight children. Ahimastos et al. [13] took pubertal-status of children into account and found significantly stiffer arteries in pre-pubertal girls compared to age-matched males, but not in post-pubertal boys and girls. Sex steroid hormones are known to influence vessel structure and function. Hence, authors hypothesize a modulating effect of male and female sex steroids, causing a reduction in arterial stiffness in girls and an increased stiffness in boys during puberty. This may result in non-significantly different post-pubertal stiffness measures. For aortic PWV, Hidvegi et al. [27] have shown a steep increase during puberty in boys and girls, which starts about 2 years earlier in girls than in boys. This earlier development in girls and presence of obesity might be the reason for significantly higher stiffness measures in obese girls in our study and identifies them as being at higher risk for early signs of atherosclerosis. Though, results of our study support a sex specific approach to arterial distensibility with obese girls being at higher risk to develop early atherosclerosis.

Equivalent to aforementioned results, cIMT and AC were not significantly correlated to BMI, whereas arterial stiffness parameters (Ep, β and PWV β) were positively associated with BMI. This reveals Ep, β and PWV β as more sensitive parameters regarding overweight and obesity in children. An increased stiffness represents a functional impairment of the arterial wall, which occurs earlier than structural alterations, defined by an increased cIMT [28]. Increased arterial stiffness is present in children with familial hypercholesterolemia, diabetes, and severe obesity [29–31]. Of all risk factors, the most obvious in the invisible atherosclerotic process is the growing number of overweight and obese children [32].

## Conclusion

Detected early in life – by one single risk factor named obesity – children can immediately improve their vascular health by losing weight [8, 12]. A reduction in BMI slows the yearly increase in cIMT and improves CVD risk factors [33, 34]. Juonala et al. [35] reported age-appropriate cIMT values in normal weight adults, who had been obese in youth but lost overweight in adulthood. This study shows a significant association between BMI and parameters of arterial stiffness, especially in girls. As obesity tracks from child- into adulthood [36], with a strong association between childhood BMI and CVD in adulthood [2, 7, 37], it is of highest priority to start prevention as early as possible. Our results support a sex-based approach to prevention programmes, as obese girls are at higher risk than normal weight controls.

## Abbreviations

β: stiffness index β
AC: arterial compliance
BMI: body-mass-index
BP: blood pressure
CCA: common carotid artery
cIMT: carotid intima-media thickness
CV: coefficient of variation
Ep: elastic modulus
PWV β: local pulse wave velocity β
SDS: standard deviation scores
